# Polyelectrolyte Matrices in the Modulation of Intermolecular Electrostatic Interactions for Amorphous Solid Dispersions: A Comprehensive Review

**DOI:** 10.3390/pharmaceutics13091467

**Published:** 2021-09-14

**Authors:** Anastasia Tsiaxerli, Anna Karagianni, Andreas Ouranidis, Kyriakos Kachrimanis

**Affiliations:** 1 Department of Pharmaceutical Technology, School of Pharmacy, Aristotle University of Thessaloniki, 54124 Thessaloniki, Greece; anastsia@pharm.auth.gr (A.T.); karagiak@pharm.auth.gr (A.K.); ouranidis@cheng.auth.gr (A.O.); 2 Department of Chemical Engineering, Aristotle University of Thessaloniki, 54124 Thessaloniki, Greece

**Keywords:** amorphous solid dispersions, polyelectrolyte matrices, interpolyelectrolyte complex, hot-melt extrusion

## Abstract

Polyelectrolyte polymers have been widely used in the pharmaceutical field as excipients to facilitate various drug delivery systems. Polyelectrolytes have been used to modulate the electrostatic environment and enhance favorable interactions between the drug and the polymer in amorphous solid dispersions (ASDs) prepared mainly by hot-melt extrusion. Polyelectrolytes have been used alone, or in combination with nonionic polymers as interpolyelectrolyte complexes, or after the addition of small molecular additives. They were found to enhance physical stability by favoring stabilizing intermolecular interactions, as well as to exert an antiplasticizing effect. Moreover, they not only enhance drug dissolution, but they have also been used for maintaining supersaturation, especially in the case of weakly basic drugs that tend to precipitate in the intestine. Additional uses include controlled and/or targeted drug release with enhanced physical stability and ease of preparation via novel continuous processes. Polyelectrolyte matrices, used along with scalable manufacturing methods in accordance with green chemistry principles, emerge as an attractive viable alternative for the preparation of ASDs with improved physical stability and biopharmaceutic performance.

## 1. Introduction

Over the past decades, novel drug delivery systems have been a key area of research into overcoming the limitations resulting from the development of poorly water-soluble drugs. The number of active pharmaceutical ingredients (APIs) having high therapeutic potential, but low water solubility, is constantly increasing. The low dissolution and bioavailability of APIs limit the available formulation approaches, their clinical application, and their marketability [1,2]. Various formulation approaches have been utilized to overcome poor biopharmaceutical drug performance, such as the creation of prodrugs, cyclodextrin (CD) complexation, various nanotechnology techniques (nanocrystals, nanoemulsions, solid lipid particles etc.), liposomes, polymorphs, hydrates, cocrystals, and others [3,4,5,6]. Among these strategies, the amorphous solid dispersion (ASD) method, where the drug is stabilized in the amorphous phase as it is incorporated into a polymeric matrix, is considered to be among the most desirable systems because of its high potential for solubility and bioavailability enhancement [3,7,8,9].

ASDs are usually prepared with the aid of fusion- or solvent-based methods. The most applicable manufacturing methods for the formation of stable ASDs are, nowadays, hot-melt extrusion (HME), and spray drying (SD), as they provide the potential for easily scalable production [10]. HME is, however, the preferred option in pharmaceutical industrial development, as it is a dust- and solvent-free method and is suitable for both batch and continuous processing [11]. Even though contemporary research has focused on the optimized production of ASDs, the selection of suitable polymeric carriers to hinder recrystallization has been mainly empirical, based on experimental trial and error approaches [12]. Furthermore, the number of polymers that are currently used in marketed ASD-based dosage forms remains limited. Therefore, a topic of growing interest is the in-depth understanding of key polymer functionalities based on physicochemical and structural properties [13]. In order to accelerate the design and formulation development process, the relationship between functional groups within a polymer for favorable intermolecular interactions, and the ability of a polymer to achieve stable ASD formulations, are currently under investigation [14,15].

Specific and nonspecific intermolecular interactions between the drug and the polymer in ASDs stabilize the preparation, and include ionic interaction, hydrogen bonding, dipole–dipole interaction, or the van der waals interaction. The presence of strong drug-polymer interactions in the system has been extensively investigated in order to promote ASD formation featuring good miscibility, drug loading, physical stability, supersaturation capability, and dissolution rate, and it is thought to facilitate the manufacturing process by fine-tuning key product parameters [15,16]. The uses for polyelectrolytes and polyelectrolyte complexes (PECs) are a field of increasing interest for ASD pharmaceutical applications, as they can promote strong intermolecular interactions of an electrostatic nature within the system. In addition, they present high biocompatibility and lower toxicity compared to other polymeric carriers, and they have been reported to facilitate the release and dissolution of the drug [17,18]. Polyelectrolytes are defined as polymers that contain a negative or positive charge at near neutral pH, whereas the term, “polyelectrolyte complexes (PECs)”, refers to complexes that form because of electrostatic interactions between oppositely charged poly-ions [19]. The interactions and/or complexation include mainly polymer-polymer, polymer-additive, polymer-drug, and polymer-drug-polymer systems. Strong electrostatic attraction can occur in these systems without the use of chemical cross-linking agents. The absence of these reagents decreases the possibility of toxic, and other undesirable, effects, making polyelectrolytes and PECs good candidates for the development of new carrier matrices used in ASD [20].

The aim of this review, therefore, is to discuss the potential of ASD-based formulations, and to highlight the potential advantages of the use of polyelectrolytes in ASDs in terms of stability, dissolution enhancement, the maintenance of supersaturation levels, and controlled/targeted drug delivery, providing a focused insight into colon-targeting preparations. Furthermore, this review thoroughly presents the applicability of the HME technique in the continuous, efficient, economical, and scalable formulation of ASDs, while modified polyelectrolyte matrices, and recently reported interpolyelectrolyte complexes as potential carriers for ASD formation, are extensively discussed.

## 2. General Principles of Amorphous Solid Dispersions

ASD is a major class of solid dispersions where the drug exists in the amorphous state and is stabilized while molecularly dispersed (dissolved) in an amorphous polymer carrier. Chiou and Riegelman were the first to define the general term, “solid dispersions”, as the “dispersions of one or more active ingredients in an inert carrier in solid form prepared by fusion, solvent or melt-solvent methods” [21]. As a rule, the AΡΙ in solid dispersions may be dispersed as individual particles in the amorphous or crystalline state, while the carrier may be in a crystalline or amorphous state [22,23]. Specifically, ASDs can be subdivided into amorphous solid (or glass) solutions, and amorphous solid (or glass) suspensions, depending on the physical state of the drug. Amorphous solid solutions are considered single-phase systems, in which the drug and the amorphous carrier can be fully mixed homogeneously, and the drug particles are dispersed as individual molecules in the carrier [11,24]. On the other hand, in amorphous solid suspensions, two phases coexist as the drug presents limited solubility and, therefore, small amorphous drug particles are dispersed in the amorphous carrier [25]. Nowadays, the term “solid dispersion” is mainly linked to amorphous solid solutions of poorly soluble drugs in amorphous polymer carriers with a high glass transition temperature (Tg), as this type is commonly encountered in most recently marketed drug dosage forms [26,27].

**Figure 1:**
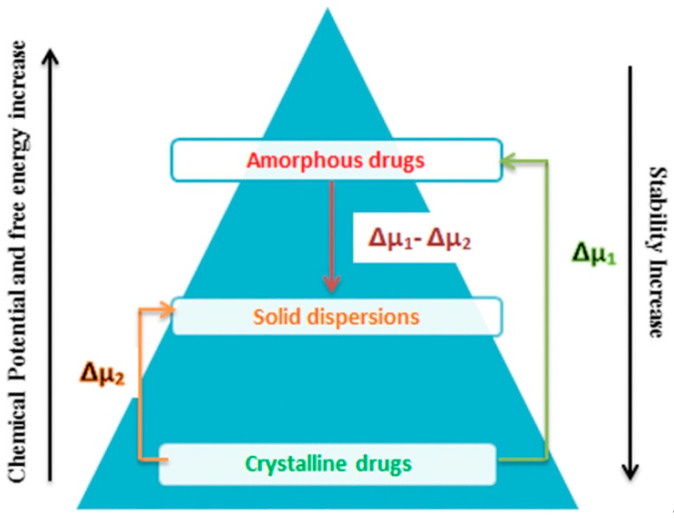
Energetic state pyramid of the crystalline phase, amorphous solid dispersion, and amorphous phase, where μ denotes the chemical potential. (Adapted with permission from [33], published by John Wiley & Sons, Inc., 2016).

Nowadays, ASDs are considered to be one of the most advantageous formulation approaches for tailoring, and providing, the superior biopharmaceutical properties of poorly soluble drugs without altering the chemical structure of the API [12]. Numerous in vivo studies have shown the enhanced dissolution rate and bioavailability profile of ASDs [28,29,30,31,32]. Their enhanced bioavailability is a result of the collaborating effects of thermodynamic and kinetic properties. In terms of thermodynamics, there are lower energy barriers to dissolution, as the API is in the high-energy amorphous state due to the disorder of the crystalline lattice. In terms of kinetics, the reversible phase transition to the crystalline form is inhibited by the polymeric matrix, which reduces the chemical potential of the amorphous drug, as is depicted in Figure 1 [33]. The polymer interacts with the molecules of the API through intermolecular forces varying in strength (e.g., hydrogen bonds, van der Waals, electrostatic etc.), or simply by steric hindrance, thus restricting the mobility that is necessary for diffusion and aggregation, which are prerequisites for nucleation [34]. Furthermore, due to the reduction of drug particles to single molecules, the drug dissolution stage is skipped, resulting in faster drug release upon contact with the biological fluids. Moreover, the wettability is also enhanced due to the use of hydrophilic polymers that, in turn, facilitate rapid matrix hydration and further enhance drug release [35]. Apart from increasing the solubility of the API, the polymeric carrier may improve the physical stability of the solid state by reducing the molecular mobility, and increasing the glass transition temperature (Tg), as well as the viscosity, of the solid dispersion. Additionally, the intermolecular interactions between the drug and the carrier inhibit the aggregation of the particles during dissolution and, as a result, the drug is released and maintained in a constant supersaturation state that facilitates homogeneous and, therefore, accelerated absorption. Even in the case of drug precipitation caused by supersaturation in the dissolution medium, the decreased size of the particles still allows for a satisfactory dissolution rate [9].

However, despite the increased research interest, the number of ASDs available on the market is still limited [27,36]. ASDs pose many challenges, ranging from their physical and chemical instability during the manufacturing process (mechanical stress), and storage (temperature, humidity), to the limitations of transfer to large-scale production, and the difficulties in establishing in vitro/in vivo correlations due to postrelease drug precipitation in the gastrointestinal tract [5,37]. Current research efforts are focusing on better understanding ASD performance, and facilitating or improving the preformulation, formulation, and manufacturing steps towards successful production. Many recent studies have contributed to this aim by: introducing novel manufacturing [38,39] and characterization techniques [22]; improving the pharmacokinetic prediction models [40]; evaluating new polymeric derivatives [14,41] and modified polymer matrices [42]; suggesting computational models for the rational selection of appropriate polymers [43] and for the prediction of the ASD crucial interactions and properties [44]; or by rationalizing the intermolecular interactions between the drug and the polymer and assessing their influence on ASD performance [15,45].

## 3. Pharmaceutical Polyelectrolytes

Over the years, different polyelectrolytes have been employed as excipients in pharmaceutical technology. Anionic polyelectrolytes are characterized by the presence of a carboxylic, sulfate, or phosphate ionizable group, with the derivatives of acrylic acid (PAA, carbomer, Carbopol), alginic acid, sodium carboxymethylcellulose (NaCMC), and hyaluronic acid. On the contrary, cationic polyelectrolytes possess a protonated nitrogen atom in their structure. Representative examples of this category are chitosan and methacrylate copolymer Eudragit E100 [46,47].

**Figure 2:**
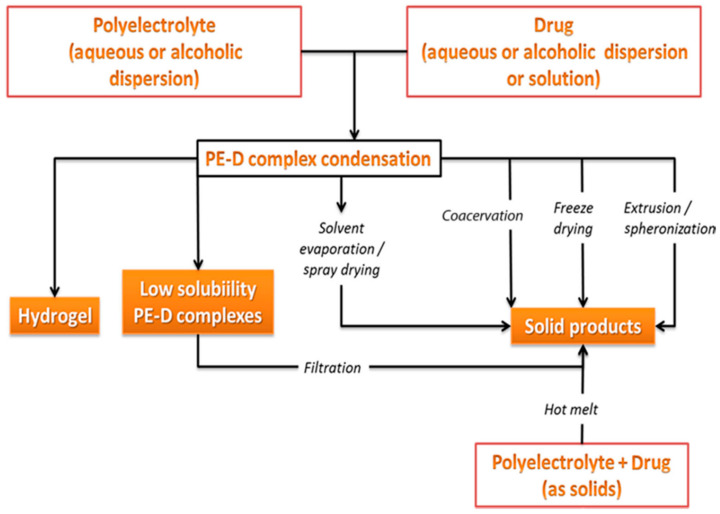
Reported processes for preparation of polyelectrolyte-drug formulations. (Adapted with permission from [46], Elsevier, Inc., 2017).

The applications of polyelectrolyte polymers are wide-ranging and they include hydrogels [48] and swellable drug carriers [49], microcapsules [50], coating membranes [51], systems for sustained or controlled/targeted drug release [52,53] as well as matrices for peptide, protein, or nucleic acid delivery to the site of action [54,55,56]. Due to the remarkable bioadhesive properties that they exhibit, polyelectrolyte use has been reported in nasal formulations [57], and buccal drug delivery systems [58], while studies for vaginal and injectable formulations have also been published [59,60]. Figure 2 summarizes the methods that have been utilized in order to prepare polyelectrolyte-drug complexes, either in aqueous media or in a solid state [46].

In ASD formulation, methacrylate copolymers, marketed under the brand name Eudragit^®^, have a remarkable presence. Eudragits are synthetic polymers obtained by the polymerization of acrylic acid (prop-2-enoic; CH_2_=CHCOOH) and methacrylic acid, or their esters. These polymers have been extensively used as excipients for enteric film-coating formulations, sustained release, taste/smell masking, and moisture prevention coatings [61,62]. Among cellulosic derivatives, anionic hypromellose acetate succinate (HPMCAS) and hypromellose phthalate (HPMCP) have been extensively used as enteric coating agents and, in recent years, have been widely studied for their applicability in ASDs [63,64]. Polyacrylic acid derivatives (Carbopol) and PVP-VA have also been reported as carriers in ASDs [65,66,67]. The various polyelectrolytes used in ASD formulations, together with the corre-sponding method of preparation, are summarized in Table 1.

## 4. Polyelectrolytes in Hot-Melt Extrusion

Hot-melt extrusion (HME), as an environment friendly, low-cost, fast and continuous operation, has become a pivotal process in ASD formulation in order to achieve enhancement in drug dissolution rates and drug release modification [96,97]. According to this method, drug and polymers in the solid state are melted and mixed together into a heated barrel with single or twin rotating screws, without the need for solvents [98]. However, the number of pharmaceutically acceptable polymers employed in HME is still limited [36]. Many efforts are currently focusing on widening the range of successfully extrudable polymers through the synthesis of novel polymers, or by the combination of the available polymers with surfactants, plasticizers, and other additives [99]. The use of polyelectrolytes, in the latter case, could enhance the potential of superior polymer matrices for HME by exploiting their capability to form molecular interactions with the API or the other excipients in the solid dispersion [100].

Several polyelectrolytes have been employed in the HME process for the preparation of stable ASDs showing an enhanced dissolution rate for various poorly soluble APIs, such as Carbopol [66], NaCMC [99], and HPMC-AS [79,81]. Among them, Eudragit is a commonly studied group of polymers that exhibits a pH-dependent biopharmaceutical behavior based on the number of acidic or alkaline end groups. Their thermoplasticity and physiochemical properties (melt viscosity, glass transition temperature, temperature stability) make them good potential candidates for use in oral ASD formulations that can be prepared by HME [61,89].

An amorphous single-phase supersaturated system comprised of the poorly soluble acidic drugs naproxen and Eudragit EPO, as well as a furosemide-Eudragit EPO polyelectrolyte complex, were successfully produced by HME [68]. The selection of substances was made by the contingent acid–base reaction in the melt. Furthermore, the influence of different inorganic salts (as electrolytes) at varying conditions was also examined in this study in order to control the drug release from the polyelectrolyte complexes, and assess their influence on the complex stability [68]. HME was also the method of choice for the successful development of amorphous solid dispersions in the case of efavirenz with Eudragit EPO [69]. Efavirenz acted as a plasticizer for the polyelectrolyte, as 50% drug loading led to a decreased viscosity in the system, enabling flow of the melt during the extrusion. Transparent extrudates were produced at 120 °C, and the occurrence of the amorphization of the dispersion was verified by DSC, PXRD, and FT-IR studies [69]. In a similar study, extrudates of nimodipine-Eudragit EPO and nimodipine-PVP/VA were successfully produced. IR data confirmed the formation of hydrogen bonding between the secondary amine group of the drug and the functional groups of polyelectrolytes, leading to higher miscibility compared to nimodipine-HPMC extrudate. The observation of homogenous extrudates, and the detection of a single Tg in these systems, elucidated the plasticizing effect of nimodipine on the polymers at the operating temperature. Results from in vitro dissolution studies revealed significant improvement in the dissolution rate of the amorphous solid dispersions of nimodipine compared to physical mixtures [70].

Eudragit EPO has also been investigated for the development of high drug loading ASDs with the model drugs indomethacin, ibuprofen, and naproxen, in maximum drug concentrations of 65, 70, and 60% *w*/*w,* respectively, at low temperatures and preoptimized conditions. Stronger drug-polymer interactions were obtained in the melt in comparison to the quench-cooling method, and no apparent recrystallization of the drugs was detected after storage at 95% relative humidity [71]. However, in the case of enalapril maleate, preparation via HME with Eudragit EPO led to the production of undesirable, potentially carcinogenic enalapril diketopiperazine because the dimethylamino groups of the EPO reacted with the acidic maleate group, especially at high temperatures [72].

**Figure 3:**
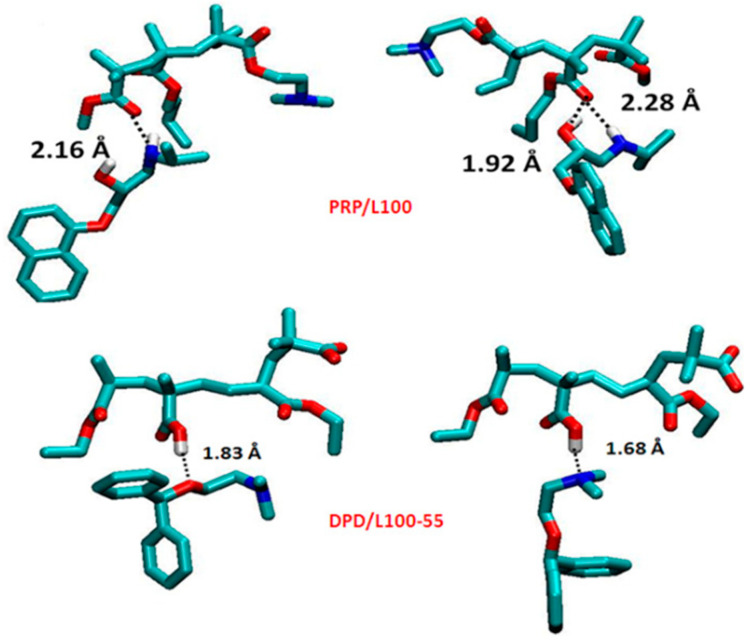
Hydrogen bonding patterns of propranolol/Eudragit L100 (PRP/L100) and diphenhydramine/L100-55 calculated by Gaussian 09. (Adapted with permission from [73], Elsevier, Inc., 2017).

Propranolol HCL and diphenhydramine HCL, as cationic model drugs, were successfully processed via HME with Eudragit L100 and Eudragit L100-55 as polyelectrolyte carriers, while thermal analysis confirmed the amorphous state of the complexes. Results from an X-ray photon spectroscopy (XPS) advanced surface analysis of the extrudates revealed the existence of hydrogen bonds between the amide groups of the drugs and the carboxyl group of polyelectrolytes, in complete agreement with the results of molecular modeling, Figure 3. Researchers also concluded that the miscibility of the drug with the polyelectrolytes defines the extent of intermolecular interactions [73]. Amorphous extrudates of metoprolol succinate were also obtained in Eudragit S100 and Eudragit L100 matrices. Addition of Eudragit L100-55 in Eudragit S100 and L100 formulations contributed to a higher dissolution performance in comparison to neat polymers, resulting in the development of extended-release dosage forms through the HME technique [74].

Except for the different Eudragit grades, anionic polymers HPMCAS, and HPMCP of various types have been reported as suitable matrices for stable ASDs produced by HME [76,79,101]. Their pH-dependent solubility has formed the basis of advanced ASD formulations in order to enhance the low bioavailability of mainly weakly ionic drugs due to the gastric pH variability [67,75,78]. This approach has been recently reported for amorphous extrudates of the weakly basic model drug nevirapine with the anionic pH-dependent soluble polymers HPMCAS, HPMCP (HP-55, HP-50), and Eudragit L 100-55, which were developed via a twin-screw extruder. Appropriate extrusion parameters were obtained and stable final formulations without crystalline fragments were produced, even though the high Tg and melt viscosity of the polymers, and the high melting point of the drug, made the procedure quite challenging. Results from the dissolution studies revealed the improved solubility and the independence of the elevated simulated gastric pH rates of ASDs, resulting in significant upgraded dissolution profiles, and subsequently enhanced in vivo absorption of the drug [75]. Cationic Eudragit EPO, anionic Eudragit L 100-55, Eudragit L 100, HPMC AS-LF, and HPMC AS-MF were processed with the weakly acidic drug indomethacin, the weakly basic drug itraconazole, and neutral griseofulvin via HME. Researchers concluded that extrudates of indomethacin with the cationic polymers, and itraconazole with anionic polymers, present a remarkable improvement in the supersaturation and dissolution rates due to the drug–polymer ionic interactions [76]. Similarly, Eudragit L 100-55 and Hydrophilic Polymer-Coated Polyurethane (HPCP) grades HP-55 and HP-55S were employed as carriers for itraconazole ASD produced by HME, leading to an improved in vivo performance due to the maintenance of the supersaturation ratio of the drug, especially in the itraconazole Eudragit L 100-55 formulation [77]. Accordingly, the ASDs of the weakly basic drug ketoconazole in the HPMCAS LG and Eudragit L100-55 matrices were produced using HME to achieve the maximum potential dissolution performance at neutral pH. ASD with Eudragit L100-55 as a carrier in a drug load of 10% was determined as the optimal formulation. However, the dissolution rate at elevated pH values was noticeably decreased compared to the static dissolution experiment at pH 6.8. This was due to phase separation, which arose during the contact of ASDs with the acidic medium, an obstacle that was circumvented with the use of enteric capsules [78].

Nifedipine-HPMCAS and efavirenz-HPMCAS amorphous extrudates were effectively developed, while FT-IR data confirmed the existence of strong intermolecular interactions. Among the different grades of the polymer, HPMCAS-MG and HPMCAS-LG for nifedipine and efavirenz, respectively, were proven to be the optimal formulations due to their capacity to achieve the higher solubility rate and the extended maintenance of supersaturation [79]. ASDs of nimodipine- HPMCAS-HF were also produced by HME after a determination of the critical process parameters. The drug-polymer intermolecular interactions, as confirmed from FT-IR studies, were found to be responsible for the maintenance of the amorphous state of nimodipine, and the inhibition of recrystallization. Furthermore, dissolution studies revealed an enhanced bioavailability rate of the ASD compared to the pure drug, and was almost equal to the market product [80]. Similarly, optimal amorphous extrudates of fenofibrate with three different grades of HPMCAS were developed and no recrystallization occurred after 45 days at accelerated conditions [102]. A recent study, in order to overcome the limitation of the high temperature required for the extrusion of HPMCAS [81], suggested different surfactants, such as poloxamer 188, poloxamer 407, and d-alpha tocopheryl polyethylene glycol 1000 succinate, to act as plasticizers and facilitate the process. Ternary solid dispersions of the model drug itraconazole with HPMCAS and surfactant were developed by HME in considerably lower temperatures and remained stable for at least one month [81]. Rheological analysis revealed that the extrudability of HPMCAS was improved significantly since the plasticization effects of the drug and the additives led to a remarkable decrease in the viscosity of the polymer [103].

Extrudates of nitrendipine with HPMCP and Carbopol as carriers were produced via twin screw extrusion. Powder X-ray diffraction and DSC analysis confirmed the amorphous state of the drug in the solid dispersions, and results from FT-IR analysis revealed the presence of hydrogen bonding between the secondary amine groups of nitrendipine, the hydroxyl groups of HPMCP, and the carboxyl groups of Carbopol, respectively. Τhe electrostatic interactions between nitrendipine and HPMCP, although proven to be stronger, decelerated the dissolution profile of the drug, making Carbopol a more suitable carrier to enhance the dissolution of nitrendipine [66]. Moreover, polyvinyl acetate phthalate (PVP-VA) was recently utilized in HME as a carrier for indomethacin ASDs. Even though the processability of the polymer was proven to be limited due to the separation of the phthalic and acetic acid fractions at elevated temperatures, successful formulations were produced with the addition of PEG 3000 as the most suitable plasticizer. Results from the dissolution studies of indomethacin PVPVA ASDs revealed a notable solubility enhancement at a pH of 5.5, in contrast to the frequently used carriers HPMCAS and Eudragit L100-55. At a pH of 6.8, the dissolution was proven to be equal for the ASDs of indomethacin with the three different polymers [67].

## 5. Modified Polyelectrolyte Matrices

### 5.1. Combination of Polyelectrolytes with Polymers

**Figure 4:**
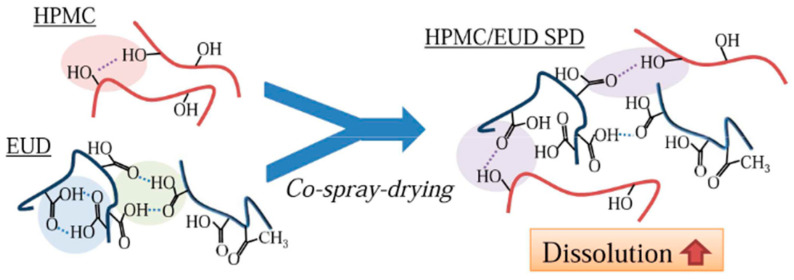
Synergistic role of Eudragit and HPMC in dissolution enhancement. (Adapted with permission from [104], Elsevier, Inc., 2017).

The concept of combining polyelectrolytes with other polymers to optimize the dissolution profile and the resistance toward the crystallization of amorphous drug formulations has been reported in several studies. Table 2 summarizes successful cases of polyelectrolyte combinations together with the API used, and achieved advantage. The synergistic role of Eudragit^®^ L100 or Eudragit^®^ S100 with hypromellose (HPMC) was investigated via co-spray drying, Figure 4. The improved dissolution profile of the polymeric blend was attributed to the intermolecular association between the hydroxyl group of HPMC and the carboxylic group of Eudragit, thus hindering the formation of intramolecular interactions [104]. Furthermore, an increased supersaturation level of griseofulvin was observed after dissolution studies of griseofulvin/HPMC/Eudragit amorphous solid dispersions, compared to the binary mixtures of griseofulvin/HPMC, and griseofulvin/Eudragit, respectively [104].

In many studies, polyelectrolytes have been combined effectively with other polymers in order to optimize the HME processability or the stability of the extruded ASDs [105,106]. A polymeric blend of HPMCAS-HF with Soluplus as a carrier successfully enhanced the physical stability of amorphous high-loading carbamazepine extrudates. FT-IR data confirmed the intensive hydrogen bonding interaction by the presence of HPMCAS-HF acting synergistically with Soluplus, leading to the molecular stabilization of carbamazepine within the ASD. Furthermore, the hydrophobic nature of HPMCAS-HF, and the high glass transition temperature, prevented the ASD of extensive water uptake and the crystallization of the API upon storage [105]. Correspondingly, a combination of Eudragit EPO and Soluplus in an equal weight ratio facilitated the extrusion of carbamazepine below its melting temperature, leading to high drug loading amorphous extrudates with a residual number of microcrystalline phases. The extrudates maintained their physicochemical stability for over three months, and the dissolution rate presented remarkable improvement [106].

The incorporation of Carbopol 974P as a stabilizing agent into a Eudragit L100-55 HME-matrix containing itraconazole had a positive effect on supersaturation levels. The strong association of the drug and the polymers, and the maintenance of this electrostatic interaction in the solution, were apparently prolonged by increasing the viscosity of the acidic polymer, resulting in delayed precipitation and, thus, a prolongation of supersaturation [107]. In another study, a combination of Eudragit E and neutral Eudragit NE in ASDs of felodipine revealed that the presence of Eudragit NE altered the structural composition of Eudragit E, resulting in an excess increase in the dissolution rate of felodipine, and a minimized recrystallization tendency [108].

### 5.2. Interpolyelectrolyte Complexes

The use of interpolyelectrolyte complexes (IPECs), obtained as precipitates upon mixing polyelectrolytes with an opposite charge in aqueous media, has been reported as an effective approach to broaden the applicable polymers in oral drug delivery systems, and they could possibly be used as matrices in ASDs [109,110,111], Table 3. IPECs can be divided into two main classes: stoichiometric, where the ratio of polymers is 1:1, and nonstoichiometric IPECs, with the additional amount of one polyelectrolyte [112]. In general, IPEC formation includes three crucial main steps: a primary complex formed rapidly, mainly by coulomb forces, followed by intramolecular complexation with the formation of new bonds and alterations in the polymeric chains, and, finally, the intercomplex aggregation of the secondary complexes is obtained, mainly through hydrophobic interactions [113,114]. Various parameters have been reported to influence the stability and formation of IPECs, including: the degree of ionization, the density of the charges and the charge distribution over the polymeric chains, the concentration of the polyelectrolytes, the mixing ratio of the polymers and the mixing order, the duration of the interaction, the nature and position of the ionic groups on the polymeric chains, the molecular weight of the polyelectrolytes, the polymer chain flexibility, as well as the temperature, ionic strength, and the pH of the reaction medium [115,116,117,118]. An extensive report on the already employed IPECs is being attempted in this section of the review, while IPECs for colonic drug targeting are described in detail in a specific section below.

**Figure 5:**
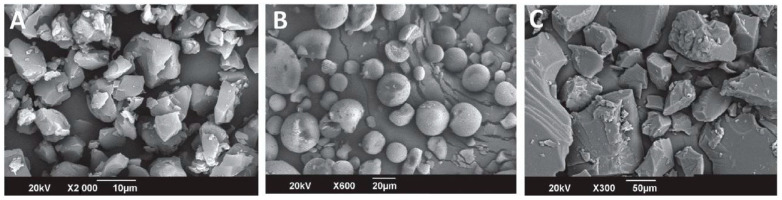
SEM images of (**A**) amorphous Eudragit EPO, (**B**) spherical Eudragit L100, (**C**) amorphous stoichiometric IPEC of EPO/L100. (Adapted with permission from [119], Scientific Electronic Library Online, Brazil, 2018).

An IPEC between cationic Eudragit EPO and anionic Eudragit L100 was successfully developed via the solvent evaporation method at a pH of 6.0, in which both polyelectrolytes were soluble and partially ionized, Figure 5. FT-IR spectra of the synthesized polycomplex revealed the existence of electrostatic interactions between the ionized functional groups of L100 and the protonated dimethylamino-groups of EPO. The binding ratio of the two polymers in the synthesized IPEC was found to be 1:1, according to the results of an elemental analysis (C/N ratio), which were reasonably close to the results of the turbidity and viscosity measurements [109]. The IPEC was additionally investigated at three different pH values (6.0, 6.5, and 7). Elemental analysis (C/N ratio) revealed that the incorporated fraction of Eudragit EPO into the complex increased as a function of the pH with a twofold excess compared to L100 in the higher pH value. Monitoring of the microenvironmental structural alterations of the new matrices during swelling in the simulated gastrointestinal fluids showed that all IPECs could potentially be used as pH-sensitive carriers because of structural and compositional transformations [110]. Additionally, Montaña et al. [119] successfully developed the same interpolyelectrolyte complex and investigated the ability of using it as a controlled release matrix for dexibuprofen. The results of the DSC thermograms revealed the presence of a single Tg at 80 °C, indicative of the miscibility of the polymers due to electrostatic interactions, while SEM images confirmed the amorphous state of the complex. Furthermore, the existence of ionized groups in the IPEC was considered responsible for its high hygroscopicity compared to the neat polymers. Dissolution profiles of dexibuprofen revealed that the IPEC enabled a controlled release of the API for 4 h, due to the formation of hydrogen bonds and the electrostatic and hydrophobic interactions, making this complex a potential candidate for smart drug delivery systems [119].

Following the same synthesis process of mixing polyelectrolyte solutions, Mustafine et al. [120] formed interpolyelectrolyte matrices of Eudragit EPO with Eudragit L100-55 at a pH of 5.5. The FT-IR spectra of the complex at a molar weight ratio of 1:1 exhibited notable alterations compared to those of a physical mixture of the two polymers due to the ionic interactions between the carboxylate groups of L100-55 with the protonated dimethylamino groups of EPO. The existence of electrostatic interactions between the two polyelectrolytes in the novel matrix was confirmed by MT-DSC thermograms that exhibited a single Tg, which significantly deviated from that calculated by the Gordon–Taylor equation. As an alternate to avoid the time-consuming procedure of mixing polymeric solutions, interpolyelectrolyte matrices of Eudragit EPO with HPMC-AS, and Eudragit EPO with HPMC-phthalate, were successfully developed in situ in an acidic medium simulating gastric fluid. Tablets of EPO-HPMCAS and EPO-HPMCP at a 1:1 weight ratio exposed at a pH of 6.8 in a phosphate buffer in a dissolution apparatus at 37 °C for 2 h, and the resulting solid complexes, were filtered and dried at room temperature. The results of the FT-IR analysis of the newly formed matrices confirmed the interaction between the carboxylic group of anionic polymers and the dimethylamino groups of cationic Eudragit EPO. The drug release of acetaminophen from the novel IPECs was effectively prolonged over 12 h, depending on the mixing ratio, the concentrations, the pH conditions, and the polyelectrolyte combination type [121].

**Figure 6:**
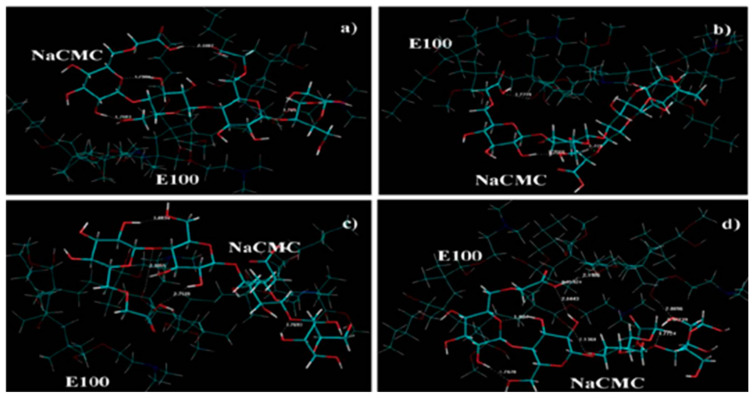
Molecular mechanic simulations of the synthetic process of NaCMC/E100 IPEC. (**a**) initial stage of mixing, (**b**) after 1 h, (**c**) breaking point, (**d**) final formation of the IPEC. (Adapted with permission from [122], MDPI, 2013).

In their study, Ngwuluka et al. [122] elucidated the rheological and mechanical alterations that took place during the formation of a hybrid Eudragit E100-NaCMC IPEC. A computational simulation of the process (Figure 6) revealed that only intramolecular bonds in NaCMC were detected at the onset of the synthesis. Intermolecular interactions between the two polymers were obtained after 1 h of the synthesis, and the formation of a consistent matrix finally led to the formation of several intra- and inter-molecular bonds. Furthermore, an acceleration of the synthesis was observed experimentally as the normality of acetic acid was increased from 0.1 to 0.4 and 0.8, respectively, with a decrease in the final point of the process from 3 h to 1 h. The new IPEC presented advantages in terms of the swelling degree compared to neat NaCMC, leading to extended gastric retention and, subsequently, controlled release of levodopa over time at a continuous rate, making it also suitable for the oral drug delivery system.

### 5.3. Addition of Small Molecules

As suitable pharmaceutical polymers for the development of ASDs are limited, research has focused on the development of modified polyelectrolyte matrices combining the already used polymers with small molecular additives [125,126]. From this point of view, ternary solid dispersions of probucol, Eudragit EPO, and saccharin were formed by cryogenic grinding. Electrostatic interactions between the amide group of saccharin and the tertiary amino group of the polymer were reported, while the preservation of the amorphous state was attributed to the hydrophobic interactions between the drug and Eudragit EPO. In addition, the dissolution profile of probucol in neutral solutions was remarkably enhanced due to these intermolecular interactions, in comparison to the binary mixtures without saccharin [125].

**Figure 7:**
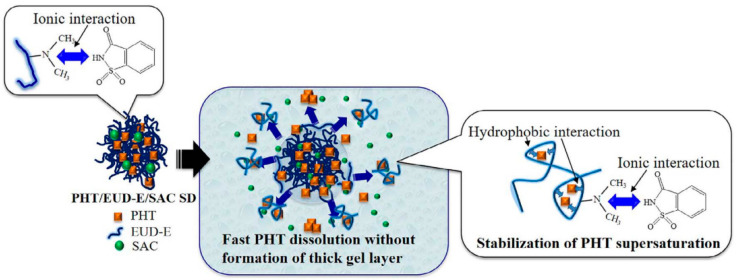
Schematic interpretation of phenytoin dissolution and supersaturated solution formed by phenytoin: Eudragit EPO: saccharin ASDs. (Adapted with permission from [126], Elsevier, Inc., 2018).

Accordingly, the addition of saccharin to the phenytoin-Eudragit EPO amorphous system accelerated the dissolution rate and improved the supersaturation level of the drug, due to the ionic interactions between the polyelectrolyte and the additive molecule. The molecular interactions between Eudragit EPO and saccharin were proven to be responsible for the promotion of the dissolution of EPO from the gel layer, resulting in the enhancement of the dissolution and the supersaturation rate of the drug (Figure 7). At the same time, the supersaturated state of phenytoin was maintained by hydrophobic interactions with the polymer [126].

Ditzinger et al. [100] designed a novel system based on the molecular interactions between cationic Eudragit E and maleic acid after being coextruded. A homogenous glassy solution was produced via HME. FT-IR and NMR analyses confirmed the interaction between the tertiary amine of Eudragit E and the carboxylic group of the coformer. The ASD of fenofibrate with the new nonchemically modified polyelectrolyte matrix showed an upgraded extrusion profile compared to the fenofibrate-Eudragit E ASDs, with improved distribution of the drug into the matrix and quick resolidification after the extrusion. An advantageous impact of this formulation was also reported in terms of the physical stability and the drug release profile during the dissolution studies. In order to overcome the drawbacks of neat NaCMC in HME, modified polyelectrolyte complexes were developed by the same team, with the addition of small molecules. The modification of the matrix was conducted through a combination process, which included an initial solvent evaporation step and subsequent HME. The selection of additive molecules was based on ionic interactions with the polyelectrolyte. Among the different interacting excipients that were studied, the addition of lysine and meglumine in the optimal amounts led to fully amorphous homogenous NaCMC matrices with enhanced extrusion behavior [99]. In vitro dissolution studies conducted on these systems with fenofibrate as a model drug, and further pharmacokinetic evaluation in rats, revealed a significantly improved bioavailability profile compared to the physical mixtures. Particularly, the extrudates of the NaCMC-lysine amorphous complex presented superior enhancement in maximum concentration and maintenance of the supersaturation of fenofibrate, leading to subsequent faster and more effective absorption of the drug [42]. In both the aforementioned cases, the target of molecular interactions manipulation was predominantly between the polymer and the additive rather than the drug. A list of modified polyelectrolyte matrices and corresponding APIs and advantages achieved, is given in Table 4.

## 6. Physical Stability due to Intermolecular Interactions

The physical stability of the amorphous drug remains one of the most challenging issues during the formulation, development, and storage of an ASD [127]. Among other factors, a noticeable enhancement in the physical stability of ASDs in terms of aging and recrystallization, or phase separation, could be achieved when strong intermolecular interactions, i.e., ionic interactions between the drug and the polymer, are present. These drug-polymer interactions efficiently reduce the molecular mobility of the drug in ASDs, and have been considered as a key kinetic factor for enhanced physical stability [128]. The potential for long-term stability because of delayed onset and the reduced extent of drug crystallization is related to the strength of such intermolecular interactions between the components of an ASD. Since ionic interactions are much stronger than H-bonds, thus suggesting greater stability, several studies have focused on ionic interactions in the ASDs of polyelectrolyte complexes [129].

Wegiel et al. [82] investigated the physical stability of amorphous curcumin dispersions with Eudragit E100, and the role of curcumin–polymer intermolecular interactions, in delaying crystallization. They concluded that electrostatic drug–polymer interactions between the acidic phenol hydroxyls of curcumin and the amino groups of Eudragit E100 appear to be the reason for the stability of the amorphous system towards crystallization, since little evidence of curcumin–polymer hydrogen bonding was monitored by mid-IR spectroscopy. The same group has also highlighted the lack of hydrogen bonding in amorphous naringenin and quercetin solid dispersions with Eudragit E100. Mid-IR spectroscopy results revealed the ionic interactions of the dimethylamino group of E100 with the polyphenols, leading to increased long-term physical stability [83]. Analysis in naproxen- Eudragit EPO and furosemide-Eudragit EPO ASDs with molecular spectroscopy methods (FT-IR, Raman) revealed ionic interactions in the formed polyelectrolyte complexes of the amorphous single phase melt [68]. Compared to naproxen sodium salt, naproxen- Eudragit EPO dispersion showed enhanced moisture resistance during storage. In XRPD measurements, no structural changes were observed, with stability being attributed to the electrostatic interactions within the complex. Therefore, in terms of long-term storage, the formation of ASDs with polyelectrolytes appears to be a viable alternative for overcoming the constraints resulting from the salt formation of poorly soluble drugs [130].

Stability studies of the ASDs of lapatinib with acidic HPMCAS and HPMCP revealed that the drug remained in the amorphous state for over six months, even after storage at accelerated conditions. The enhanced physical stability of the two systems was attributed to the drug-polymer intermolecular interactions. In the case of lapatinib-HPMCP, remarkably higher Tg values than those predicted indicated a strong intermolecular interaction. Solid-state NMR analysis elucidated the formation of ionic interactions between protonated lapatinib within the phthalate groups of the polymer, while the stabilization of lapatinib-HPMCAS was possibly due to hydrogen bonding. In the same study, PVPVA and nonionic Soluplus were characterized as poor crystallization inhibitors since they failed to maintain the stability of the amorphous dispersions of lapatinib [84]. The presence of ionic interactions was also reported in the case of ketoconazole-PAA ASDs. The strong drug-molecular bonding consequently led the system to a dramatically decreased molecular mobility, and decreased the possibility of fast recrystallization, even at low polymer concentration. Furthermore, an increase in relaxation time was reported as a function of the polymer concentration at a given temperature [85].

**Figure 8:**
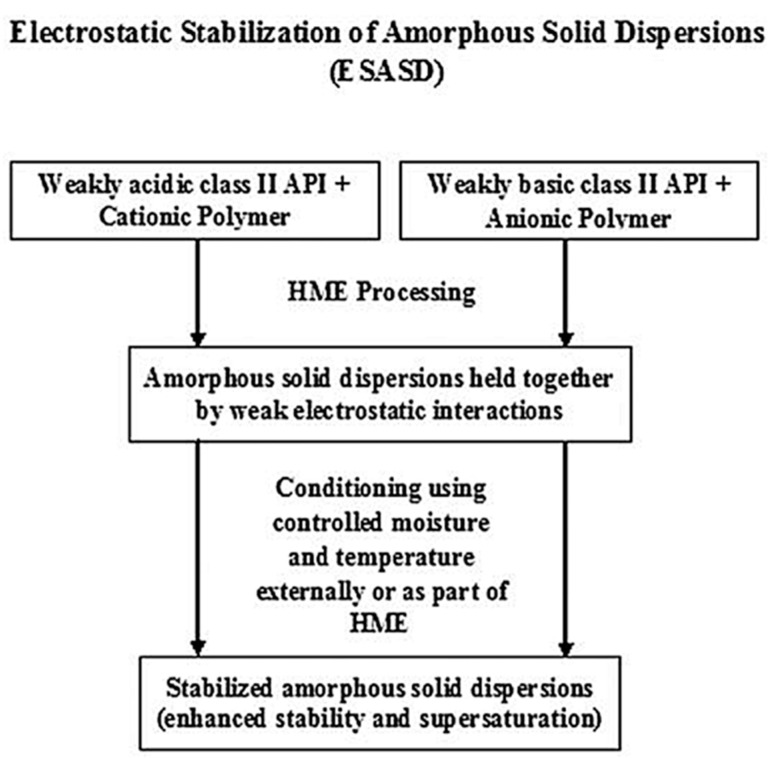
Electrostatic stabilization of amorphous solid dispersions of polyelectrolytes under controlled moisture and temperature conditions. (Adapted with permission from [86], American Chemical Society, 2013).

In another study, an unusual instance of the enhancement of electrostatic interactions under appropriate temperature and humidity conditions was observed in the amorphous extrudates of indomethacin, Eudragit EPO and itraconazole, and HPMCAS-LF, after three months of storage. Sarode et al. [86] suggested that water could act as the catalyst or medium to support the charge transfer between the counterionic moieties, resulting in the electrostatic stabilization of ASD, optimized physical stability of the extrudates, and the supersaturation of the drug during dissolution, as it is represented in Figure 8.

### The Antiplasticization Effect of Polyelectrolytes

The antiplasticization phenomenon, known as the reduction in the plasticity of a material, is evident by the increase in the Tg of the drug in an ASD while the free energy for its recrystallization increases [33]. The antiplasticization effect can result in the improved stability of ASDs during storage and, in many cases, may provide a good indication of strong intermolecular interactions. For instance, in the case of indomethacin and Eudragit E ASDs, measured Tg values were greater than the predicted ones, indicating strong ionic interactions, which were finally confirmed by highly specific solid-state NMR spectroscopy [87]. In a similar study, researchers concluded that the stable ASD that was formed between indomethacin and Eudragit EPO, and the higher Tg than the value calculated from the Gordon–Taylor equation for the system, were attributed to ionic intermolecular interactions that prevented intramolecular interactions and drug dimer formation [88]. Accordingly, the IR-data of PAA-loperamide solid dispersions elucidated that electrostatic forces between the amino groups of the drug and the acid groups of the polymer exist and, as a result, the Tg-values of the dispersions increased, leading to inhibition of the crystallization of the fragment molecules during preparation [65]. However, antiplasticization can itself result in the enhanced stability of ASDs, as in the case of Eudragit EPO in efavirenz ASD. Antiplasticization was assumed to be the key factor responsible for the prolonged physical stability of the amorphous extrudates, even though no strong intermolecular interactions were observed in the FT-IR analysis [69].

## 7. The Impact of Polyelectrolytes on the Dissolution Rate and Supersaturation

The extent and maintenance of supersaturation achieved by polyelectrolyte ASDs are crucial parameters to be considered, as they directly affect the dissolution and absorption properties of active pharmaceutical ingredients that suffer from poor bioavailability [131,132]. The induced metastable supersaturated solution can effectively improve the drug bioavailability by an increase in the free drug concentration and the thermodynamic driving force for oral absorption [133]. Polyelectrolytes in ASDs accomplish this by inhibiting the drug precipitation of the supersaturated solution for a sufficient period of time, thus providing an elevated and sustained drug supersaturation state [134]. Among many water-soluble polymers that are commonly used in marketed ASD-based drug dosage forms, the polyelectrolyte HPMCAS has been reported as one of the most widely used polymers, showing the highest effectiveness in achieving and maintaining drug supersaturation [36,135]. Recently, the maximum attainable concentration of the ASDs of lopinavir with different polyelectrolytes has been shown to vary with the extent of drug-polymer interactions [89]. Correspondingly, the remarkable maintenance of the supersaturation of itraconazole in a neutral pH solution was attributed to the existence of acidic functional groups on the polymer chain of Eudragit L 100-55, and the strength of the intermolecular interactions between the drug and the polymer [77].

Studies on a supersaturated solution produced by mefenamic acid-Eudragit EPO amorphous solid dispersion revealed that drug-polyelectrolyte intermolecular interactions are responsible for the high stabilization of this system. The maintenance of supersaturation was attributed to both the hydrophilic and hydrophobic interactions between the carboxyl group of mefenamic acid and the tertiary amine of the EPO, and the two aromatic groups of the drug with the backbone of the polymer, respectively, as demonstrated in the 2D1H/1H NOESY and RFDR MAS NMR analyses [90]. Results from the dissolution profile of the mefenamic acid-EPO amorphous system in acetate buffer (pH 5.5) indicated an excessive increase in the drug concentration and, consequently, an enhanced oral bioavailability when the supersaturated solution was administered to rats. Solubility improvement and the stabilization of the supersaturated solutions for over a month in acetate buffer were also reported in the dissolution tests of the piroxicam-Eudragit EPO and the indomethacin-Eudragit EPO amorphous systems. Thus, researchers concluded that solid dispersions of cationic EPO with anionic drugs could advance drug solubility, and the maintenance of supersaturation and oral bioavailability, due to electrostatic interactions in the system [17].

Beak and Kim [18] investigated the relationship between supersaturation behavior, measured during dissolution, and the in vivo absorption enhancement of dutasteride formed with Eudagit E via the spray drying process. The results indicated that solid dispersion with Eudragit E displays a high maximum and extended supersaturation compared with other polymers, such as HPMC, HPC, and PVP. After oral administration in rats, the AUC_0__→24 h_ and C_max_ of dutasteride increased with the supersaturation concentration. Similarly, the ASDs of sirolimus with Eudragit E were efficiently manufactured by the spray drying method. The evaluation of the dissolution studies showed that the dispersion significantly inhibits the degradation and precipitation of sirolimus in a dose-dependent manner. The addition of a nonionic polymeric surfactant, such as D-α-tocopheryl polyethylene glycol 1000 succinate (TPGS), to the formulation enhanced the stability and degree of supersaturation, as shown by the dissolution and solubility tests in simulated gastric fluid, while pharmacokinetic studies in rats indicated a significant improvement in the oral absorption of sirolimus [91].

On the contrary, the dissolution studies of the ASDs of ezetimibe monohydrate with PAA in different biorelevant media revealed that crystallization from the solid matrix occurred rapidly when the ASDs came into contact with either the sodium phosphate buffer or the fed state simulated intestinal fluid medium, incapable of achieving supersaturation, while only moderate supersaturation was maintained for a longer time period in a biorelevant lipid-rich medium [92]. Similar results were observed in the dissolution profiles of celecoxib-PAA ASDs, where an extremely fast dissolution rate was exhibited at high drug loadings. However, the supersaturation of the ASDs decreased rapidly, and the drug concentration approached the crystalline solubility in a small amount of time [93]. The abovementioned studies have affirmed PAA as a poor crystallization inhibitor, despite its polyelectrolyte nature and high water solubility that facilitates a fast drug release. However, a stable ASD of the loperamide with PAA showed the suitability of this polyelectrolyte carrier in the suppression of crystallization and the increase in the dissolution rate for basic compounds [65].

## 8. Polyelectrolytes in Controlled-Drug Release/Colonic Targeting

Recently, extensive research has been conducted assessing the performance of pH-responsive, water-insoluble, and modified polyelectrolytes in controlled-release studies [136,137]. Controlled-release ASDs have been studied in order to reduce the required drug dose and, subsequently, improve patient compliance [138]. Moreover, they could reduce the incidence of undesirable systemic adverse reactions, and they have been exploited for enhanced and targeted absorption [139,140]. At this point of the review, we will focus on the well-studied colonic drug targeting as an interesting approach to enhance the therapeutic profile by delaying absorption of the drug and providing more efficient treatment for colon diseases. In colon-targeting carrier matrices, drug release takes places at a pH > 7, and is controlled either by the swelling of polymers that leads to diffusion transport, or by the gradual erosion processes of the system that finally leads to drug elution [141]. From this point of view, polyelectrolytes and novel interpolyelectrolyte complexes have been investigated as potential carriers for effective targeted drug delivery to the colon [141].

In their study, Balogh et al., [94] produced ASDs of Eudagit FS100 (EudFS), an anionic pH-dependent methacrylate terpolymer with spironolactone used as a model drug via the electrospinning technique. EudFS-based solid dispersions were also prepared via melt extrusion but, in this case, residual crystalline traces were detected. Transmission Raman spectroscopy revealed the presence of stronger polymer-drug interactions in the electrospun samples than in the extruded materials. The in vitro dissolution results confirmed the efficiency of the EudFS matrix in preventing drug release at acidic pH, and a dramatic increase in the dissolution rate was observed at a pH above 7. Another interesting study has reported the successful preparation of the ASD of berberine hydrochloride with Eudragit S100 by a solvent evaporation method to delay the release of the drug in the stomach and improve its anticancer efficacy in human colon cancer cells. The results confirmed that the electrostatic interactions between the drug and the polymer enhanced the antitumor activity, making this system a potential new candidate for colon cancer therapy [95].

A novel approach to targeting the colon involves the use of the anionic polymer Eudragit S100 to form IPECs with cationic Eudragit EPO. The results of the FT-IR spectroscopy, and the modulated-temperature differential scanning calorimetry, confirmed that the new IPEC was stabilized by the cooperative intermacromolecular ionic bonds between ionized EPO dimethylamines and the S100 carboxyl groups. The investigation of the swelling capacity in media imitating passage through the gastrointestinal tract pointed out the increasing swelling of the polycomplex structural fragments, and the subsequent compaction in the final medium with a pH = 7.4, corresponding to the characteristics for a carrier providing delivery to the colon [123]. Additionally, the researchers elucidated the suitability of this carrier by preparing ASDs of indomethacin and Eudragit EPO/S100 IPECs at a fixed pH, and investigating drug release profiles under gastrointestinal tract-mimicking conditions. The presence of Eudragit S100 in the structure was proven to provide resistance in drug release at a gastric pH and a continuous drug release profile above a pH of 6.8, where most of the carboxyl groups of indomethacin are deprotonated, while the sequences of S100 were still insoluble. This investigation proposed the application of precisely pH-controlled drug-interpolyelectrolyte ternary systems for the colon-targeting of the encapsulated drugs [111].

Similarly, a new ionic-bonded polycomplex between the soluble cationic polyelectrolyte Eudragit EPO and Carbopol 940, with high swelling capacity, was synthesized and examined as a controlled-release drug delivery system [142]. Dissolution studies of ibuprofen indicated that the characteristics of drug release from the matrix occurred due to the possible binding of ionized ibuprofen with Eudragit EPO freely oriented in the outer layer of the IPEC [124]. Further biopharmaceutical assessment confirmed that pharmacokinetic parameters of the new IPEC loaded with diclofenac sodium were comparable to those of the brand name drug Voltaren^®^ Retard, making this particular IPEC promising as a candidate for colon-specific delivery [143].

## 9. Conclusions

As our understanding of the drug-polymer interactions responsible for the stabilization of the amorphous state is increasing, the natural next step in the development of ASDs is the modulation of the aforementioned interactions by the clever choice of materials possessing chemical moieties that are expected to interact favorably. This rationale is of particular interest in the case of weakly basic drugs that tend to precipitate in the intestine, losing their solubility advantage. In this perspective, the use of polyelectrolyte polymers has gained much attention in the pharmaceutical industry in the attempt to extend the polymeric excipient landscape for oral drug formulations. The application of polyelectrolytes and novel polyelectrolyte complexes as matrices for ASDs could lead to more effective delivery systems with controlled and targeted drug release, while the intermolecular interactions could enhance the bioperformance and the physical stability of the ASDs. ASDs based on polyelectrolyte polymers and their complexes constitute a promising enabling technology for the maintenance of supersaturation in biological fluids, especially in the case of weakly basic drugs. Scalable manufacturing methods, such as hot-melt extrusion, could be utilized in accordance with green chemistry principles to investigate innovative interpolyelectrolyte complexes and modified polyelectrolyte matrices for further improvement in drug delivery applications.

On the downside, only a limited number of polyelectrolyte polymers has proven suitable for processing by hot-melt extrusion, a fact that is limiting the available choices for the formulation scientist. Newly investigated polymers, such as NaCMC, or novel materials, such as interpolyelectrolyte complexes, cannot be easily formulated into ASD matrices. One additional limitation regarding their physical stability enhancement and supersaturation-maintaining effect, is that they are mainly effective in the case of weakly acidic/basic APIs, where the electrostatic and polarization interactions are more prominent.

In our opinion, future research should focus on the rational model-based selection of suitable polyelectrolye polymers. Both thermodynamic (Flory-Huggins, Hansen Solubility Parameter, PC-SAFT, etc.), and atomistic modeling (molecular dynamics simulations), have a lot to offer as a means for the interpretation of experimental findings and the prediction of suitable polymers for API categories. As our understanding of the main types of interactions responsible for API crystallization advances, atomistic simulations, especially, can be of grave importance to ASD formulation design, in general. Another field that needs to be more intensively researched relates to the HME development process relevant to polyelectrolyte polymers. As already discussed, many promising polyelectrolyte polymers have shown poor processibility alone. Therefore, there is a need for research on suitable additives that will not impair, but rather enhance, their beneficial action on supersaturation maintenance, in addition to the enhancement of their processibility.

In conclusion, although the use of polyelectrolytes is not a general solution to ASD stability and biopharmaceutical performance problems considering its limitations, nevertheless, despite the aforementioned limitations, it represents yet another step forward in our efforts to control critical factors affecting physical stability and drug release and could, eventually, lead to ASD-based pharmaceutical products of enhanced safety and efficacy.

## Figures and Tables

**Table 1:** Use of polyelectrolytes in ASDs.

API	Polyelectrolyte Matrix	Preparation Method	ASDs	Ref.
Naproxen, Furosemide	Eudragit EPO	Hot-melt extrusion	Existence of ionic interactions in the melt	[68]
Efavirenz	Eudragit EPO	Hot-melt extrusion	Plasticization effect of efavirenz/Facilitation of extrusion	[69]
Nimodipine	Eudragit EPO, PVP/VA	Hot-melt extrusion	Higher miscibility compared to nonionic polymers, due to intermolecular interaction	[70]
Indomethacin, Ibuprofen, Naproxen	Eudragit EPO	Hot-melt extrusion	High drug-loading amorphous extrudates/Strong intermolecular interactions	[71]
Enalapril maleate	Eudragit EPO	Hot-melt extrusion	Formation of carcinogen enalapril diketopiperazine at high temperatures	[72]
Propranolol HCL, Diphenhydramine HCL	Eudragit L 100, Eudragit L 100/55	Hot-melt extrusion	Intermolecular interactions between the amide groups of the drugs and carboxyl group of polyelectrolytes	[73]
Metoprolol succinate	Eudragit S 100, Eudragit L 100	Hot-melt extrusion	Advanced dissolution performance with addition of Eudragit L100-55 to the extrudates	[74]
Nevirapine	HPMCAS, HPMCP (HP-55, HP-50), Eudragit L 100-55	Hot-melt extrusion	Stable final formulations without crystalline fragments with improved in vivo absorption	[75]
Indomethacin, Itraconazole, Grizeofulvin	Eudragit EPO Eudragit L 100-55, Eudragit L 100, HPMC AS-LF and HPMC AS-MF	Hot-melt extrusion	improved supersaturation and dissolution levels due to drug–polymer ionic interactions	[76]
Itraconazole	Eudragit L 100-55, HPCP (HP-55 and HP-55S grades)	Hot-melt extrusion	Maintenance of supersaturation of the amorphous extrudates	[77]
Ketoconazole	HPMCAS LG and Eudragit L100-55	Hot-melt extrusion	ASDs with Eudragit L100-55 in a drug load of 10% the optimal formulation	[78]
Nifedipine, Efavirenz	HPMCAS grades	Hot-melt extrusion	Strong intermolecular interactions in the melt	[79]
Nimodipine	HPMCAS-HF	Hot-melt extrusion	High physical stability due to intermolecular interactions	[80]
Itraconazole	HPMCAS	Hot-melt extrusion	Improvement of extrusion with the addition of poloxamer 188, poloxamer 407 and d-alpha tocopheryl polyethylene glycol 1000 succinate as plasticizers	[81]
Nitrendipine	HPMCP, Carbopol	Hot-melt extrusion	Deceleration of dissolution due to electrostatic interactions between nitrendipine and HPMCP/Carbopol more suitable	[66]
Indomethacin	PVP-VA	Hot-melt extrusion	Amorphous extrudates with addition of PEG 3000 as plasticizer	[67]
Curcumin	Eudragit EPO	Solvent evaporation, cryo-milling	Enhanced physical stability due to electrostatic drug–polymer interactions	[82,83]
Lapatinib	HPMCAS, HPMCP	Spray drying	Stabilization of the ASD due to ionic interactions between protonated lapatinib with the phthalate groups of the polymer	[84]
Ketoconazole	PAA	Solvent evaporation and melt-quenching	Intermolecular interactions led to inhibition of recrystallization	[85]
Indomethacin, Itraconazole	Eudragit EPO, HPMCAS-LF	Hot-melt extrusion	Electrostatic stabilization of ASDs	[86]
Indomethacin	Eudragit EPO	Spray drying	Strong ionic interactions in the matrix	[87,88]
Loperamide	PAA	Spray drying	Inhibition of recrystallization	[65]
Lopinavir	HPMCAS, HPMCP	Solvent evaporation	Variation in the extent of ASD concentration depend on drug-polymer interactions	[89]
Mefenamic acid	Eudragit EPO	Cryogenic grinding	Maintenance of supersaturation in solution due to electrostatic interactions	[17,90]
Dutasteride	Eudragit E	Spray drying	Extended supersaturation compared to ASDs with nonionic polymers	[18]
Sirolimus	Eudragit E	Spray drying	Enhancement in physical stability and dissolution profile with addition of TPGS	[91]
Ezetimibe monohydrate	PAA	Solvent evaporation	Fast crystallization in sodium acetate buffer	[92]
Celecoxib	PAA	Solvent evaporation	Rapid decrease of supersaturation of ASDs with high drug loading	[93]
Spironolactone	Eudragit FS100	Electrospinning/Hot-melt extrusion	Stronger polymer-drug interactions in the electrospun fibers than extrudates	[94]
Berberine Hydrochloride	Eudragit S100	Solvent evaporation	Enhancement in antitumor activity due to electrostatic interactions	[95]

**Table 2:** Advantages of the synergistic role of combination polyelectrolytes with nonionic polymers.

Polyelectrolyte	Nonionic Polymer	API	Advantage of Synergistic Role	Ref.
Eudragit L100, Eudragit S100	HPMC	Griseofulvin	Improvement of dissolution profile/Increase in supersaturation level of the API	[104]
HPMCAS-HF	Soluplus	Carbamazepine	Molecular stabilization of carbamazepine in the amorphous extrudates	[105]
Eudragit EPO	Soluplus	Carbamazepine	Extrusion of carbamazepine below its melting point/Enhanced physicochemical stability of the ASD	[106]
Eudragit L100/55	Carbopol 974P	Itraconazole	Delayed precipitation and improvement of supersaturation	[107]
Eudragit E	Eudragit NE	Felodipine	Enhancement of dissolution rate/Prevention of recrystallization	[108]

**Table 3:** List of reported IPECs and the intermolecular interactions in their formation.

Anionic Polyelectrolytes	Cationic Polyelectrolytes	IPEC Preparation Method	Intermolecular Interactions	Ref.
Eudragit L100	Eudragit EPO	Solvent evaporation at pH 6.0, 6.5, 7.0	Ionized functional groups of L100 with the protonated dimethylamino-groups of EPO	[109,110,119]
Eudragit L100/55	Eudragit EPO	Solvent evaporation at pH 5.5	Carboxylate groups of L100-55 with the protonated dimethylamino groups of EPO	[120]
HPMC-AS/HPMCP	Eudragit EPO	Dissolution method at pH 6.8 (in situ)	Carboxylic group of HPMCAS and HPMCP with the dimethylamino groups of EPO	[121]
NaCMC	Eudragit E100	Solvent evaporation	Functional groups of NaCMC with dimethylamino groups of E100 after 1 h of the synthesis	[122]
Eudragit S 100	Eudragit EPO	Solvent evaporation	S 100 carboxylic groups with the dimethylamino groups of EPO	[123]
Carbopol 940P	Eudragit EPO	Solvent evaporation	carboxyl groups in C940 with protonated dimethylamino groups in EPO	[124]

**Table 4:** Advantages of modified polyelectrolyte matrices.

Polyelectrolyte	Molecular Additive	API	Preparation Method	Advantage of Synergistic Role	Ref.
Eudragit EPO	Saccharin	Probucol	Cryogenic grinding	Enhanced dissolution profile due to intermolecular interactions compared to binary mixtures without additive	[125]
Eudragit EPO	Saccharin	Phenytoin	Cryogenic grinding	Improved supersaturation level and dissolution profile of phenytoin due to ionic and hydrophobic interactions in the matrix	[126]
Eudragit E	Maleic acid	Fenofibrate	Hot-melt extrusion	Facilitation of extrusion/ Advanced physical stability of amorphous extrudates and drug release profile	[100]
NaCMC	Lysine/Meglumine	Fenofibrate	Solvent evaporation—Hot-melt extrusion	Fully amorphous NaCMC extrudates	[42,99]

## Data Availability

Not applicable.
